# Ophthalmic Treatments and Evolving Management of Sjögren’s Disease: An Overview

**DOI:** 10.3390/jcm15187272

**Published:** 2026-09-18

**Authors:** María García Forestier, José Nassar Badillo, Armando L. Oliver

**Affiliations:** Department of Ophthalmology, School of Medicine, University of Puerto Rico, San Juan 00936-5067, Puerto Rico

**Keywords:** Sjögren’s disease, aqueous-deficient dry eye, Keratoconjunctivitis Sicca, scleral contact lenses, meibomian gland dysfunction, ocular surface inflammation

## Abstract

**Background**: Sjögren’s disease (SjD) causes severe ocular surface desiccation, progressing from aqueous-deficient dry eye (ADDE) to vision-threatening corneal ulceration, stromal melting, and perforation. Recent consensus guidelines and therapeutic innovations have transformed ophthalmic diagnosis and management. **Methods**: A comprehensive narrative overview of PubMed and Google Scholar (July 2016–July2026) was conducted to evaluate updates in SjD diagnostics, ocular surface mechanics, topical/biological pharmacotherapies, neurostimulation, and meibomian gland interventions. **Results**: Diagnostic parameters have shifted towards non-invasive salivary metabolomics (lactate, alanine, malate), regulatory non-coding RNAs (H19 ICR hypomethylation), tear point-of-care MMP-9 testing, and salivary gland ultrasonography (SGUS). Large-diameter scleral lenses provide continuous corneal fluid shielding, though edge design and surface modification are required to mitigate midday fogging. Additionally, emerging therapeutics are shifting care from palliative lubrication to targeted secretomotor and biological reactivation, including cold thermosensory TRPM8 agonists (acoltremon 0.003%), water-free formulations (0.1% cyclosporine, perfluorohexyloctane), reactive aldehyde species (RASP) modulators (reproxalap), syndecan-1 receptor agonists (Lacripep), systemic B-cell depleting biotherapy (ianalumab), trigeminal neurostimulation, and combined intense pulsed light with meibomian gland expression (IPL + MGX). **Conclusions**: Ophthalmic SjD management has evolved from palliative lubrication toward a stepwise, mechanism-directed paradigm integrating novel diagnostics, secretomotor stimulation, mechanical protection, and biologic disease modification; dedicated SjD-specific trials remain essential to validate these emerging interventions.

## 1. Introduction

Commonly known as Sjögren’s Disease (SjD), this systemic autoimmune disease attacks healthy tissue and organs, most notably the lacrimal and salivary glands that keep our eyes and mouth lubricated [1]. Clinically, this condition is now diagnosed as Sjögren’s disease without a strict split into primary and secondary variants [2]. When it overlaps with a systemic connective tissue condition, the modern consensus states that “associated” should be used over “secondary” [2]. Under this updated classification framework, the disease frequently coincides with systemic lupus erythematosus, rheumatoid arthritis, or systemic sclerosis, while being less commonly associated with multiple sclerosis, thyroiditis, and autoimmune hepatitis [3]. Regardless of clinical classification, the pathology operates as a chronic autoimmune epithelitis that targets and destroys the exocrine glands of the ocular surface unit [4].

The definitive histopathological hallmark driving the failure of lacrimal glands in Sjögren’s disease is a progressive infiltration of lymphocytes, causing periductal and perivascular inflammation [3]. This chronic pathogenesis is led by infiltrating CD4+ helper T cells. These cells differentiate into distinct Th1, Th2, and Th17 subsets, causing widespread tissue destruction through localized cytokine cascades [3,5]. Simultaneously, abnormally activated B cells rapidly multiply and differentiate into plasmablasts and mature plasma cells, creating an abundance of tissue-targeting autoantibodies [5]. As this autoimmune dacryoadenitis advances, cytotoxic CD8+ T cells and memory B cells secrete inflammatory cytokines, including IFN-γ and TNF-α, promoting the apoptosis of secretory acinar cells and causing severe ductal destruction [3,5]. Over time, this targeted immune response permanently changes the functional structure of the gland, causing the parenchyma to be replaced by extensive fibrosis and adipose tissue accumulation [3,4]. Without this reflex, the cornea is left unprotected against epithelial degradation and remains at high risk for irreversible structural damage [3,4].

Additionally, this localized inflammatory response also produces a secretomotor neuroinnervation injury, in which pro-inflammatory cytokines and autoantibodies impair neurotransmitter action, paralyzing the normal corneal-trigeminal reflex loop and blocking fluid output [3]. By blocking the secretion and damaging the lacrimal unit, Sjögren’s disease causes Aqueous-Deficient Dry Eye (ADDE) [6]. If left untreated, this deficiency in tear production causes a rapid hyperosmolar desiccation across the ocular surface epithelia, forcing clinicians to focus on preventing irreversible tissue degradation [7]. The ophthalmic presentation of Sjögren’s disease represents one of the most aggressive forms of ocular desiccation, with the potential to transition from a simple surface dryness to severe, vision-threatening tissue degradation [8]. This progressive destruction can manifest clinically as severe corneal ulcers, localized stromal melting, and corneal perforations that can lead to permanent visual impairment [8].

Fortunately, therapeutic options have evolved significantly in recent years, introducing advanced interventions such as large-diameter scleral lenses to enclose the compromised cornea in a moist environment, promoting active epithelial healing [9]. New pharmacotherapies also focus on neurosensory agonists, such as acoltremon 0.003% (Tryptyr; ID: NCT07277257), which stimulates cold thermosensory neurons and directly activates the lacrimal unit to restore baseline tear production, reducing ocular damage and symptoms of dry eye [10,11]. Therefore, this electronic narrative overview evaluates the clinical updates from 2016 to 2026 regarding ophthalmic diagnostic evaluation, systemic disease staging, and modern therapeutic interventions for ophthalmic symptoms of Sjögren’s disease, providing clinicians with an updated framework to preserve long-term visual function.

## 2. Materials and Methods

### 2.1. Search Strategy and Data Sources

A structured electronic narrative overview was conducted to evaluate updates in diagnostic testing, staging, and therapeutic methods regarding ophthalmic manifestations of Sjögren’s disease. Literature queries were executed across the National Institute of Health (NIH) PubMed Search and Google Scholar databases from inception to 25 July 2026. The search architecture relied on Medical Subject Headings (MeSH), abstract/title keywords, and was structured as follows: (“Sjögren’s Disease” OR “Sjögren’s Syndrome” OR “autoimmune dacryoadenitis” OR “sicca syndrome”) AND (“ocular surface inflammation” OR “corneal melt” OR “aqueous-deficient dry eye” OR “meibomian gland dysfunction” OR “keratoconjuctivitis sicca” OR “tear film instability”) AND (“cyclosporine” OR “scleral lenses” OR “neuromodulation” OR “TRPM8 agonist” OR “acoltremon” OR “ianalumab” OR “intense pulsed light” OR “thermal pulsation” OR “perfluorohexyloctane” OR “salivary gland ultrasound” OR “proteomics”). To ensure comprehensive capture of emerging therapies and regulatory updates, electronic database searches were supplemented by hand-searching reference lists of included consensus guidelines, systematic reviews, Clinical Trial registry entries (e.g., THALASSA trial [ID: NCT07621809]), and official US Food and Drug Administration (FDA) approval documentation.

### 2.2. Inclusion and Exclusion Criteria

To include the most precise data, the search scope was restricted according to the following parameters. Inclusion criteria included: (1) randomized controlled trials (RCTs), prospective and retrospective cohort studies, case series, case reports, and systematic reviews; (2) studies evaluating human subjects with Sjögren’s disease, including those with associated systemic autoimmune conditions (e.g., SLE, RA), presenting with aqueous-deficient dry eye or associated corneal pathology; (3) peer-reviewed publications in the English language within the designated 10-year timeline (July 2016–July 2026). Exclusion criteria included: (1) abstract-only publications, non-peer-reviewed papers, and non-English manuscripts without official translations; (2) studies focusing on non-Sjögren’s dry eye phenomenon (e.g., contact lens-induced dryness or age-related meibomian gland dysfunction) without a clear clinical translation or relevance to the autoimmune pathology and ocular surface mechanics of Sjögren’s disease; (3) studies lacking direct clinical translation or correlation to human ocular tissue.

### 2.3. Data Extraction and Synthesis

Data extraction was performed independently across all retrieved publications. Extracted parameters included study design, participant sample size, clinical trial phase (if applicable), specific study population characteristics (e.g., exclusive Sjögren’s disease (SjD) cohorts from generalized dry eye disease or meibomian gland dysfunction cohorts), primary diagnostic or therapeutic mechanism, ocular surface metrics (e.g., Schirmer test, tear breakup time, corneal/conjunctival staining scores), patient-recorded symptom outcomes (e.g., Ocular Surface Disease Index, SANDE, visual analog scale), and reported adverse events. The synthesis was structured to address: (1) updated diagnostic criteria, biomarkers, and imaging; (2) mechanical fluid shielding via scleral lenses and amniotic membranes; (3) topical and biological pharmacotherapies; (4) neurostimulation and systemic biotherapies; (5) photothermal interventions. Findings from non-SjD cohorts were explicitly evaluated for mechanistic translatability to SjD exocrine pathology.

To address potential selection bias and account for extrapolation of evidence from broad dry eye populations, extracted interventions were categorized into three distinct evidence tiers: Category 1 (Direct SjD Clinical Evidence; studies conducted exclusively in SjD cohorts); Category 2 (Mixed/Subgroup Evidence; studies or meta-analyses evaluating pooled populations with explicit SjD subgroup analyses); and Category 3 (General DED/Laboratory Extrapolation; studies conducted in generalized dry eye, meibomian gland dysfunction, or in vitro tissue models where utility in SjD is mechanistically extrapolated).

## 3. Results

### 3.1. Advanced Diagnostic Methods and Criteria

#### 3.1.1. Clinical Workup and Disease Nomenclature

Historically, classification relied on invasive procedures, such as salivary gland biopsies and serological testing [12]. The clinical approach to confirming and diagnosing Sjögren’s disease has shifted away from sole reliance on patient history-taking and towards non-invasive biomarkers, standardized ocular surface metrics, and advanced tissue imaging [12,13]. Because ophthalmologists are frequently the first clinicians to encounter these patients due to the key presenting symptoms of unexplained dry eye, understanding both updated systemic classification criteria and standardized ocular diagnostic tools is critical for guiding early referrals and further workups [14]. An international consensus officially changed the disease nomenclature from “Sjögren’s Syndrome” to Sjögren’s Disease (SjD). This update established the condition as a distinct, systemic biological disorder rather than an “ill-defined” cluster of symptoms [2]. Additionally, by retiring the primary versus secondary variants in favor of a singular, comprehensive pathology, the consensus simplifies the actual diagnostic workflow for the clinician [2]. Under these updated guidelines, when SjD overlaps with a connective tissue disorder, such as rheumatoid arthritis or systemic lupus erythematosus, the updated criteria designate the condition as an “associated” rather than a “secondary” variant, since the underlying autoimmune mechanics works the same on polyautoimmunity [2].

#### 3.1.2. Objective Ocular Metrics, Point-of-Care Testing, and Molecular Diagnostics

Ultimately, a definitive diagnosis requires testing both oral and lacrimal units simultaneously [13]. In the eye clinic, objective quantification of aqueous tear volume begins with unanesthetized Schirmer test strips, where a score of ≤5 mm in 5 min indicates severe lacrimal gland failure [7]. To evaluate ocular surface structural integrity, clinicians use fluorescein staining to locate any corneal epithelial erosion under cobalt blue light, and lissamine green or rose bengal dye stains to visualize damaged epithelium on the conjunctiva [13]. These staining patterns are routinely quantified using standardized scoring frameworks, such as the National Eye Institute (NEI) grid, where elevated staining scores reflect epithelial damage driven by chronic hyperosmolar desiccation [6,15]. Tear film stability is evaluated via tear breakup time, with rapid breakup (<10 s) highlighting severe tear volatility [16]. Modern molecular diagnostics are also improving eye clinic workflows by analyzing tear film proteomics and exosomal biomarkers [17]. For instance, point-of-care tear testing allows for rapid, in-office quantification of elevated matrix metalloproteinases-9 (MMP-9) and pro-inflammatory cytokines, allowing ophthalmologists to detect active ocular surface inflammation cascades much earlier than historical protocols allowed [17,18]. In an effort to achieve earlier and more precise diagnostic methods, multi-omics research from October 2014 to November 2024 identified a wide spectrum of non-invasive salivary biomarkers [12]. This systematic review combined a review of the last decade of research to evaluate changes in salivary diagnostics. For instance, metabolomic profiling of saliva demonstrates increased concentrations of metabolites, such as lactate, alanine, and malate, which are directly correlated to severe chronic hypoxia, tissue inflammation, and active oxidative stress within the glandular epithelium [12]. Furthermore, molecular screening of regulatory non-coding RNAs has isolated specific alterations, such as hypomethylation of the imprinting control region (ICR) of the H19 gene locus, upregulation of microRNA let-7i-5p, and downregulation of miR-17-5p [12].

#### 3.1.3. Glandular Ultrasonography, Biopsy, and Lymphoma Screening

Non-invasive imaging has become an established tool to evaluate salivary gland structural abnormalities [13]. Salivary Gland Ultrasound (SGUS) can be an alternative to open biopsy, using the Outcome Measures in Rheumatology Clinical Trials (OMERACT) semi-quantitative 0–3 scoring system to evaluate tissue based on the presence and density of hypoechoic areas and hyperechoic fibrous bands [13,19]. In patients demonstrating advanced structural tissue damage matching a score of 2 or 3, progressive gland swelling raises high suspicion of mucosa-associated lymphoid tissue (MALT) non-Hodgkin’s lymphoma, the most severe long-term complication of Sjögren’s disease [13]. When assessing lymphoma risk, clinicians must closely identify parotid gland swelling with systemic laboratory signs, as evidence of leukopenia, palpable purpura, and low serum complement C4 levels indicate extremely high risk for oncologic transformation [16]. Additionally, ultrasound-guided core-needle biopsy (CNB) of the posterocaudal parotid gland has been described as a safe, highly accurate diagnostic approach, providing viable tissue to evaluate histopathological markers without the surgical risk seen in open tissue resection biopsies [13]. In contrast to the frequent swelling seen via ultrasound of the major salivary glands, macroscopic enlargement of the lacrimal glands remains relatively uncommon in patients presenting with SjD [17]. Consequently, when a clinical enlargement of the lacrimal gland is seen on examination, clinicians must immediately start a diagnostic workup to rule out overlapping infiltrative diseases, including lymphoma, sarcoidosis, amyloidosis, and IgG4-related diseases [17].

### 3.2. Standard-of-Care and Conventional Treatment Framework

The clinical management of Sjögren’s disease (SjD)-related ocular desiccation follows a stepped-care hierarchy adapted from global dry eye management guidelines, such as the Tear Film & Ocular Surface Society Dry Eye Workshop II (TFOS DEWS II) [6,20]. Under this management framework, therapy begins in Step 1 with patient education regarding the chronic nature of the condition, environmental modifications, identifying and modifying potential exacerbating systemic medications, and the regular use of over-the-counter ocular lubricants [20].

Traditional clinical management of Sjögren’s disease (SjD)-related ocular dryness has relied on a palliative approach centered on symptom mitigation rather than disease modification [3,17,18]. For instance, preservative-free artificial lubricants, sodium hyaluronate eye drops, and viscous ointments for nighttime remain the foundational standard of care for patients with SjD to replace deficient tear volume and hydrate ocular surfaces [3,18]. When non-preserved tear substitutes are insufficient, Step 2 management escalates to conserve tears via punctal occlusions, such as silicone or collagen punctal plugs. Additionally, nocturnal moisture chamber devices and non-pharmacological eyelid hygiene protocols are also used [20].

To manage acute inflammatory flares and suppress ocular surface immune cascades, Step 2 guidelines incorporate short-term topical corticosteroids or long-term topical immunomodulatory agents, such as calcineurin inhibitors (Cyclosporine A) and integrin antagonists (Lifitegrast) [3,17,20]. However, the prolonged use of topical corticosteroids is strictly limited due to the well-documented adverse side effects, such as cataract formation and increased intraocular pressure [17]. Additionally, systemic therapies, such as pilocarpine or cevimeline are used to stimulate muscarinic cholinergic pathways, which increase fluid output across exocrine glands [3,18]. Nevertheless, because these treatments only offer transient relief without decreasing or stopping progressive autoimmune dacryoadenitis, modern ophthalmic research has shifted towards targeted, mechanism-specific interventions to restore ocular surface homeostasis [6,18].

### 3.3. Scleral Lenses and Ocular Surface Mechanics

#### 3.3.1. Aqueous Deficiency and Scleral Lenses

The progressive damage and tissue destruction of the lacrimal glands detailed above results in severe aqueous tear deficiency [17]. Within the broader framework of dry eye disease (DED), this loss of homeostatic equilibrium presents as an Aqueous-Deficient Dry Eye (ADDE) [6,15]. Left unchecked, the drop in basal tear volume exposes the interpalpebral ocular surface to severe, continuous stress, creating an environment characterized by focal tear film instability, tissue damage, local inflammation, and hyperosmolarity [6,15]. While this diagnostic classification and concept of homeostasis loss were written for the global dry eye population rather than being unique to autoimmune states, they provide a foundation for tracking how localized exocrine failure damages eyes in Sjögren’s disease (SjD) [6]. Severe DED drastically worsens a patient’s overall quality of life, matching the functional and psychological impairment observed in patients with advanced coronary artery disease or chronic skin disorders [15].

While treatment guidelines reserve specialized mechanical options for end-stage interventions (Step 3) of the treatment hierarchy of DED, there have been discussions for using large-diameter scleral lenses (SLs) earlier in the treatment [9]. SLs are large-diameter rigid gas-permeable contact lenses technically designed to cover the sensitive corneal epithelium and rest on the conjunctival tissue overlying the sclera [9,15]. By filling the lens with non-preserved saline prior to the application of the device, clinicians can keep the cornea permanently enclosed in a liquid environment [9]. This fluid barrier effectively protects the ocular surface, completely blocking any tear film evaporation and maintaining a moist environment for corneal healing [9]. It is worth noting that while contemporary meta-analyses evaluate the clinical role of these devices across generalized dry eye populations lacking primary corneal irregularities, the continuous fluid reservoir system might be highly useful for the severe desiccation profiles seen in advanced Sjögren’s disease [9]. Additionally, recent evidence highlights the use of specialized, free-form adaptive designs that replicate the unique ocular surface of each patient via digital surface profilometry [15]. Results show that when worn for at least eight hours a day, these customized lenses cause a drastic reduction in corneal National Eye Institute (NEI) scores, complete recovery of chronic epithelial defects, and improvements in best-corrected visual acuity (BCVA) [15].

#### 3.3.2. Scleral Lens Complications and Mitigation Strategies

However, implementing rigid lenses in an active Sjögren’s patient introduces specific mechanical barriers, such as trapped debris and midday fogging [15]. Up to 75% of dry eye patients using scleral lenses suffer from midday fogging (MDF), a significant complication where cellular debris, inflammatory neutrophils, and matrix metalloproteinases (MMP-9, MMP-10) get trapped in the post-lens fluid reservoir for hours and turns the saline opaque [9]. Additionally, scleral lens therapy can occasionally cause secondary complications, including corneal neovascularization, localized edema, or conjunctival prolapse, where loose conjunctival tissue folds over the cornea due to asymmetrical pressure from the lens [15]. To bypass these barriers and avoid patient dropout, modern protocols emphasize maximizing edge alignment with toric or quadrant-specific haptics, eliminating excessive fluid reservoir thickness (FRT) to minimize the accumulation of debris, and applying a 40-nanometer-thick polyethylene glycol (PEG)-based surface treatment to the lens, reducing friction and optimizing long-term comfort [9,15].

### 3.4. Targeted Topical, Neurosensory, and Biological Ophthalmic Therapeutics

#### 3.4.1. Neurosensory Activation: TRPM8 Agonists and Trigeminal Reflex Loop Stimulation

While structural shielding via scleral lenses manages the downstream effects of severe ocular desiccation, modern ophthalmic research has shifted towards therapies that can directly reactivate native, basal tear secretion at the cellular level [10]. This therapeutic goal has driven the clinical development of highly selective neurosensory agonists, most notably those targeting the transient receptor potential melastatin 8 (TRPM8) ion channel expressed on corneal cold thermosensory neurons [10]. Under normal physiological conditions, subtle shifts in environmental temperatures cool the ocular surface, activating these channels and signaling the central nervous system to trigger a homeostasis reflex arc that increases basal tear production [10]. By topically implementing a novel, potent TRPM8 chemical agonist, such as acoltremon 0.003%, clinicians can successfully bypass the damaged secretomotor pathways to stimulate the eye’s natural tearing reflex [10].

The clinical efficacy and safety of this acoltremon 0.003% (Tryptyr, Alcon) were recently tested in a pivotal, randomized Phase 3 COMET-2 and COMET-3 clinical trials, leading to its FDA approval on 28 May 2025, for signs and symptoms of dry eye disease [10,21]. In these trials, patients applying topical acoltremon achieved highly significant improvements in objective tear production across a broad dry eye population [10]. Although these definitive phase 3 registration protocols were built around general dry eye disease criteria rather than an isolated autoimmune subpopulation, the observed secretomotor activation provides a plausible mechanistic reasoning for its potential application in SjD, through dedicated clinical evaluation in autoimmune cohorts remains necessary [10]. More specifically, a large number of patients met the primary endpoint of achieving a ≥10 mm increase from baseline on the unanesthetized Schirmer test by day 14 of treatment compared to the control group [10]. Additionally, this response was followed by a reduction in subjective patient discomfort across multiple validated evaluations, including the Symptom Assessment in Dry Eye (SANDE) scale and standard visual analog scales (VAS), which evaluate burning, stinging, and dryness [10]. Acoltremon’s direct neurostimulation provides a reliable mechanism to increase baseline fluid production [10]. Because acoltremon also acts with high selectivity on low-threshold cold receptors rather than the high-threshold nociceptors that signal pain, the drug was well tolerated, demonstrating a highly favorable ocular safety profile with minimal application-site irritation [10]. However, further research is needed on the effectiveness of this neurosensory approach in patients with advanced glandular atrophy.

Additionally, device-based pathways can leverage the rich neural network of the exocrine system by stimulating the trigeminal nerve endings located within the nasal mucosa [22]. By delivering electrical stimulation to these afferent pathways, an intranasal neurostimulator device sends signals from the afferent lacrimal center in the brain to the efferent autonomic secretomotor nerves, improving the unanesthetized Schirmer test scores regardless of a patient’s baseline tear volume or medication status [22]. While this device has faced commercial discontinuation and controversies, these findings suggest that mechanical neurostimulation of the exocrine reflex arc could successfully trigger acute tear production and meibomian gland contraction for patients suffering from dry eye symptoms [22,23]. While the aggregated meta-analysis data confirming significant increases in post-stimulation tear volume evaluated a pooled sample of generalized dry eye subjects rather than an exclusive autoimmune population, the underlying trigeminal reflex arc provides a relevant physiological model for addressing secretomotor impairment in SjD [23]. Building upon this trigeminal reflex arc, recent clinical research investigated the pharmacological use of multi-dose, preservative-free varenicline solution (0.03 mg) nasal spray [24]. By acting as selective nicotinic acetylcholine receptor agonist, intranasal varenicline chemically activates the mucosal parasympathetic pathway that triggers the secretion of natural aqueous, lipid, and mucin tear film components [24]. As a result, this treatment has been shown to improve ocular surface hydration, corneal staining, and dry eye symptoms seen in Sjögren’s disease without any direct contact with the corneal epithelium [24].

#### 3.4.2. Anti-Inflammatory Solutions, Water-Free Delivery, and RASP Modulators

In addition to direct neurosensory activation of the corneal cold receptors, modern ophthalmic solutions also work by delivering water-free immunomodulators, inhibiting localized upstream inflammatory cascades, and introducing therapeutic peptides to repair cellular defects [25,26,27]. Because traditional cyclosporine eye drops often cause local intolerance due to their high lipophilicity and formulation limitations, novel delivery methods have transformed the clinical space [25]. For instance, a water-free, perfluorobutylpentane-based formula of 0.1% cyclosporine (Vevye) significantly improves drug delivery, showing statistical superiority over the control group in reducing total corneal fluorescein staining and conjunctival staining by day 29 of twice-daily application in a general dry eye cohort [25]. Earlier formulations like 0.05% cyclosporine ophthalmic solutions (Restasis) rely on highly hydrophobic emulsions and suffer from low bioavailability, ocular intolerance, and blur [25]. However, this newer water-free solution contains no water, oil, or preservatives, and its low surface tension allows for rapid ocular surface spreading [25]. This formulation, marketed as Vevye in the United States (FDA approval, May 2023), is the same product approved by the European Commission in October 2024 under the name Vevizye for moderate-to-severe dry eye disease that has not improved with tear substitutes [28]. It differs fundamentally from earlier cyclosporine products. Restasis (cyclosporine 0.05%) is an anionic oil-in-water emulsion, and Ikervis (cyclosporine 0.1%), approved in Europe in 2015 for severe keratitis in dry eye disease, is a cationic oil-in-water emulsion. Both rely on emulsified oil droplets and surfactants to carry the lipophilic drug, which contributes to instillation-site burning, transient blurring, and variable ocular bioavailability [25]. In the water-free solution, cyclosporine is dissolved directly in the semifluorinated alkane vehicle (perfluorobutylpentane), so no oil, water, surfactant, or preservative is required. The very small, low-surface-tension drop spreads rapidly over the ocular surface, which underlies the improved tolerability and corneal staining outcomes reported in the pivotal trial [25,28]. Although this water-free formulation was evaluated broadly in dry eye disease rather than an isolated autoimmune population, its bioavailability and anti-inflammatory properties offer a theoretical framework for addressing the severe ocular surface inflammation characteristic of SjD, pending future autoimmune-specific trials [25]. To complement calcineurin inhibition, clinicians frequently combine topical integrin antagonists, such as lifitegrast 0.5% [17]. These drops selectively block the binding interaction between lymphocyte function-associated antigen-1 (LFA-1) on T-cells and intercellular adhesion molecule-1 (ICAM-1) on the inflamed ocular epithelium, blocking local T-cell recruitment and weakening the lymphocytic drive in Sjögren’s disease [29].

To manage acute inflammatory flares, novel interventions such as reproxalap target upstream reactive aldehyde species (RASP) modulators. Because excess RASP molecules act as pro-inflammatory mediators that affect a variety of proteins and amplify ocular surface cascades, reproxalap chemically neutralizes these molecules and acts as an anti-inflammatory solution [26]. Controlled clinical studies in a dry eye chamber model have shown that reploxalap increases tear production and significantly lower dry eye symptoms within a 90-min exposure compared to control [26]. While these symptoms and sign reductions were evaluated in a generalized dry eye cohort, the ability of reproxalap to immediately reduce inflammatory aldehyde cascades suggests a potential therapeutic rationale for investigating reproxalap as a rescue agent during acute SjD flares [26].

#### 3.4.3. Glycosaminoglycan (GAG) Therapeutics

Glycosaminoglycans (GAG) therapeutics represent another expanding biological class that combines biomechanical lubrication and immunosuppressive components across the ocular surface [30]. Hyaluronic acid (HA), an endogenous non-sulfated GAG, is already a well-established therapeutic agent for dry eye management due to its high-water retention capacity, improved tear film break-up time and stability, and reduced discomfort across varying disease severities [30]. However, expanding research highlights that GAGs have functional effects beyond passive tear film hydration [31]. In corneal epithelial models, therapeutic GAG structures actively stimulate re-epithelization and cellular migration, upregulate proliferation and differentiation markers (such as KI67, K14, and PAX6), suppress squamous metaplasia markers (Sprr1b), and promote ocular surface barrier integrity by increasing the expression of membrane-associated mucins (MUC1 and MUC4) [31]. Additionally, GAGs regulate localized inflammation by downregulating pro-inflammatory cytokines (IL-1β, TNF-α, IL-6), decreasing matrix metalloproteinase-9 (MMP-9), and reducing leukocyte and macrophage tissue infiltration (CD45) [31].

These regenerative and anti-inflammatory properties have led to the clinical development of sulfated and multi-component GAG regimens. Chondroitin sulfate, a sulfated GAG with cell-membrane stabilizing and lubricating properties, has been utilized as an ophthalmic solution to enhance drug tolerance and ocular surface healing [32]. Clinically, compounded 0.1% cyclosporine formulated in a chondroitin sulfate emulsion achieved significant reductions in both mean Ocular Surface Disease Index (OSDI) scores and corneal fluorescein staining over a 3-month period [32]. GlicoPro, a standardizes snail-mucus-derived multimolecular complex containing sulfated and non-sulfated GAGs, scaffold proteins, and opiorphin, significantly suppressed inflammatory cytokines (IL-20, IL-1β, TNF-α, IL-6, IL-8), MMP-9, and monocyte chemoattractant protein-1 (MCP-1) in human corneal epithelial cells [33]. In a randomized controlled clinical trial (ClinicalTrials.gov: NCT06726525), an ophthalmic solution combining hydroxypropyl-methylcellulose (HPMC) with GlicoPro demonstrated significant therapeutic effects, providing enhanced symptomatic relief, increased tear film stability, and a significant reduction in corneal inflammation cell density as quantified by in vivo confocal microscopy [33].

#### 3.4.4. Anti-Evaporative Monolayers and Corneal Repair Peptides

To manage severe evaporative tear film instability seen in this disease, clinicians are also using perfluorohexyloctane ophthalmic solution (Miebo) [34]. As a non-aqueous, preservative-free drop, perfluorohexyloactane spreads rapidly across the surface to form an anti-evaporative layer in dry eye patients undergoing cataract surgery [34]. Given the anti-evaporative properties demonstrated in post-cataract dry eye, this physical monolayer solution provides a conceptual approach for managing the evaporative component often co-existing in mixed-etiology SjD tear film instability [34]. Moreover, biotherapies like Lacripep (LOS-001) are evaluating topical, lacritin-derived peptides that can stimulate cell motility and exosome biogenesis via direct interactions with syndecan-1 surface receptors [27]. The molecular pathway of this drug works by the interaction between the active fragment at the C-terminus of human lacritin used in Lacripep (LOS-001) and syndecan-1 surface receptors after the removal of heparan sulfate chains by local heparinase [27]. Once bound, this interaction triggers a process that mobilizes intracellular calcium, a chemical process that promotes cell survival, induces human corneal epithelial cell proliferation, and accelerates overall wound healing across the ocular surface [27,35]. While baseline epithelial cell proliferation profiles and calcium mechanics were mapped using generalized human corneal tissue models, these repair pathways provide an in-vitro biological foundation that warrants investigation in chronic, non-healing SjD keratopathy [27,35].

#### 3.4.5. Regenerative Therapies: Amniotic Membrane Grafts and Blood-Derived Substitutes

When ocular surface desiccation escalates to catastrophic presentations characterized by progressive stromal thinning, persistent epithelial defects, or impending corneal perforation, mechanical shielding must be enhanced with biological tissue grafts [17,36]. In clinical practice, placement of sutureless, self-retained cryopreserved amniotic membranes (CAM), such as Prokera, provide an immediate in-office barrier over the compromised cornea [17,36]. These placements grafts work by releasing endogenous growth factors and anti-inflammatory cytokines, while actively suppressing matrix metalloproteinases (MMPs), including MMP-9 [36]. As a result, by suppressing the immune response and decreasing inflammation, the application of amniotic membranes could be a viable treatment for re-epithelialization in Sjögren’s disease [36]. However, clinical evidence suggests that dry eye signs and symptoms frequently relapse approximately 25 days post-membrane application, suggesting that CAM therapy may only serve as an acute structural stabilization therapy [36].

Additionally, clinicians also rely on blood-derived biological tear substitutes to supply platelets and enhance epithelial regeneration via endogenous growth factors [17,37]. In a prospective, randomized controlled trial conducted specifically in primary Sjögren’s cohorts, both autologous serum eye drops (ASED) and platelet-rich plasma (PRP) significantly increased tear breakup time and reduced corneal epithelial staining [3,37]. By delivering key endogenous growth factors, such as epithelial growth factor, transforming growth factor-beta (TGF-β), and nerve growth factor, these factors stimulate tissue repair and wound healing, and stimulate sub-basal corneal nerve fiber regeneration in refractory Sjögren’s disease [37].

### 3.5. Systemic Disease-Modifying Biotherapies

Systemic disease-modifying therapies like ianalumab (VAY736) have also been studied due to their targeted, dual action approach of preserving exocrine tissue through the depletion of pathogenic B cell populations [5,18]. For decades, systemic management of Sjögren’s disease has been limited to palliative surface hydration or broad off-label immunosuppressants [3,18]. As a fully human monoclonal antibody directed specifically against the B-cell activating factor receptor (BAFF-R), ianalumab combines immediate antibody-dependent cellular cytotoxicity (ADCC)-mediated B-cell elimination with a complete blockage of BAFF-dependent survival loops [5,18]. This targeted biological pathway is currently undergoing global evaluation in the Phase III THALASSA Trial (ID: NCT07621809) and Phase III NEPTUNUS-1 and NEPTUNUS-2 trials to assess its long-term safety to reduce systemic symptoms and reverse exocrine gland inflammation [18,38]. While systemic disease activity improvements (ESSDAI) have been reported, whether systemic immunomodulation translates into clinically meaningful ocular surface restoration and tear production in SjD remains under active investigation.

### 3.6. Intense Pulsed Light and Meibomian Gland Management

Although Sjögren’s disease is traditionally classified as a form of aqueous-deficient dry eye, modern clinical evidence suggests a high prevalence of meibomian gland dysfunction (MGD), resulting in an aggressive mixed-mechanism ocular surface disease profile [39]. To target this destructive evaporative layer, intense pulsed light (IPL) has emerged as a non-laser, broad-spectum light therapy capable of improving eyelid margin abnormalities, decreasing apoptosis of corneal epithelial and conjunctival goblet cells, and overall improvements in vision [39]. A prospective randomized clinical trial investigated the efficacy and safety of combining three distinct sessions of IPL with sequential manual meibomian gland expression (MGX) as an official polytherapy for patients suffering from refractory Sjögren’s disease-related dry eye [39]. The treatment group demonstrated clinical improvements by weeks 12 and 15, including a drastic reduction in Ocular Surface Disease Index (OSDI) symptom score and extended non-invasive tear breakup time [39]. Mechanical clearance of the terminal ducts was also noted, enhancing meibum secretion quality and a complete reduction in baseline corneal fluorescein staining scores [39]. As a result, the properties of this type of light therapy uniquely improved objective and subjective clinical symptoms in patients with Sjögren’s disease dry eye [39].

Clinical alternatives using specialized vector-thermal pulsating systems, such as TearCare or LipiFlow, also achieved significant reductions in baseline symptom presentations by safely melting and clearing blocked terminal duct meibum [40]. While these thermal clearance platforms were validated using generalized meibomian gland dysfunction cohorts rather than exclusive autoimmune study sample, their direct physiological mechanism of enhancing tear film and improving lipid secretion highlights a potential strategy for the evaporative lipid-deficient component of SjD [40]. However, it is important to note that a complete assessment is needed to guide patient selection when using any of these advanced management strategies [39]. For example, Intense Pulsed Light therapy must be carefully evaluated if Sjögren’s disease coexists with an associated connective tissue condition such as systemic lupus erythematosus, as ultraviolet/broadband light can interact with severe systemic photosensitivity and trigger an acute cutaneous or autoimmune flare [23]. Therefore, screening for underlying systemic connective tissue is highly recommended before incorporating any mechanical light modulation into a Sjögren’s management protocol [23,39].

Diagnostic, ocular surface and systemic interventions (Table 1) and regulatory status of novel interventions and managements (Table 2).

## 4. Discussion

### 4.1. Clinical Management Hierarchy for Ophthalmic Sjogren’s Disease

**Figure 1:**
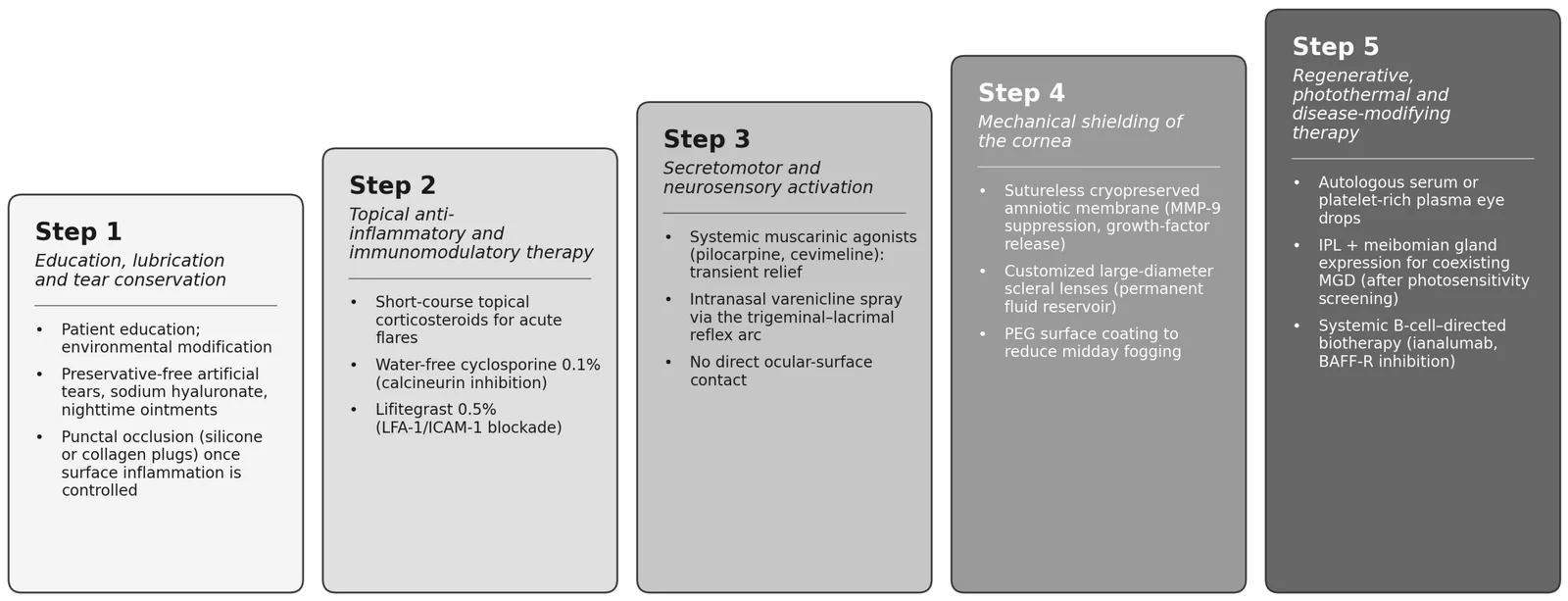
Proposed stepwise clinical management hierarchy for the ocular manifestations of Sjögren’s disease (SjD).

The management of ocular manifestations in Sjögren’s disease (SjD) demands a structured, step-by-step approach that transitions from simple symptom relief to advanced tissue protection and targeted disease modifications [18,20]. Building upon global consensus frameworks and recent clinical advancements, management follows the logical clinical hierarchy (Figure 1):

Step 1

Initial management includes patient education regarding chronic disease progression, environmental factors to minimize evaporative stress, and the frequent use of preservative-free artificial lubricants, sodium hyaluronate drops, and viscous nighttime ointments to hydrate compromised epithelia [3,18,20]. When surface tears are severely depleted, clinicians implement tear conservation strategies, such as punctal occlusion with silicone or collagen plugs, provided active surface inflammation is controlled to avoid pro-inflammatory mediators [14,20].

Step 2

To address active immune-mediated surface damage, short-course topical corticosteroids are used during acute inflammatory flares, though long-term application is strictly limited due to risks of secondary glaucoma and cataract formation [17,20]. For chronic immunodulation, newer non-preserved formulations, such as water-free 0.1% cyclosporine and integrin antagonists (lifitegrast) improve ocular surface bioavailability and block T-cell recruitment by targeting LFA-1/ICAM-1 interactions [25,29].

Step 3

Because conventional systemic muscarinic agonists, such as pilocarpine and cevimeline, only offer transient relief and fail to suppress advanced glandular fibrosis, modern care incorporates novel neurosensory pathways [3,18]. For instance, intranasal varenicline nasal spray (tyrvaya) directly stimulates the trigeminal-lacrimal reflex arc to reactivate basal aqueous tear and mucin secretion without direct ocular surface contact [22,24].

Step 4

When the disease progresses to severe epithelial breakdown or impending corneal perforation, mechanical shielding becomes essential [17]. Sutureless cryopreserved amniotic membranes (CAM) provide an acute in-office stabilizing therapy by suppressing MMP-9 and releasing growth factors, while customized large-diameter scleral lenses (SLs) enclose the cornea in a permanent fluid reservoir to promote active healing [9,15,36]. Midday fogging and debris accumulation in SL wearers could be effectively mediated via polyethylene glycol (PEG) surface coatings [9,15].

Step 5

Advanced corneal epithelial breakdown benefits from blood-derived biological tear substitutes, such as autologous serum eye drops (ASEDs) and platelet-rich plasma (PRP), which deliver growth factors that stimulate corneal re-epithelialization and sub-basal nerve regeneration [17,37]. Additionally, for patients with co-existing meibomian gland dysfunction, intense pulsed light therapy combined with meibomian gland expression (IPL + MGX) targets the evaporative component, provided the patient is screened for systemic photosensitivity [39,41]. Finally, systemic disease-modifying biotherapies like ianalumab (BAFF-R inhibition) targets the underlying autoimmune component of SjD, shifting the therapeutic goal from palliative care towards true disease modification [5,18].

### 4.2. Knowledge Gaps, Study Limitations, and Future Direction

A critical methodological limitation identified in this 10-year narrative review is the reliance on data extrapolated from generalized dry eye disease (DED) or meibomian gland dysfunction (MGD) cohorts rather than exclusive Sjögren’s disease (SjD) autoimmune populations. As structured in our evidence synthesis, several major therapeutic classes, such as cold thermosensory TRPM8 agonists (acoltremon), water-free 0.1% cyclosporine formulations, upstream reactive aldehyde species (RASP) modulators (reproxalap), non-aqueous anti-evaporative monolayer solutions (perfluorohexyloctane), and intranasal nicotinic acetylcholine receptor agonists (varenicline), were evaluated and registered primarily through broad generalized DED trials (Category 3 Evidence).

Although these novel pharmacotherapies offer compelling theoretical and mechanistic frameworks for addressing ocular surface inflammation, tear volatility, and secretomotor deficits, their true clinical efficacy in active Sjögren’s disease (SjD) remains to be tested and proven. SjD-related aqueous-deficient dry eye is driven by aggressive periductal lymphocytic infiltration, permanent acinar destruction, and glandular fibrosis. As a result, the pathological mechanisms unique to SjD, such as elevated baseline pro-inflammatory cytokines (TNF-α, IL-1β, IL-6, IL-17, and MMP-9) on the ocular surface could alter drug bioavailability, tolerance, and therapeutic response over non-autoimmune DED conditions.

Furthermore, significant knowledge gaps remain regarding the long-term clinical translation of systemic immunomodulatory biotherapies. While phase III trials investigating B-cell activating factors receptor (BAFF-R) inhibition (ianalumab) demonstrate promising systemic disease activity reduction (ESSDAI), whether profound systemic B-cell depletion successfully halts progressive corneal desiccation or restores baseline tear production is currently under active investigation. As a result, future ophthalmic research must prioritize dedicated, disease-specific Category 1 clinical trials conducted exclusively in SjD cohorts to validate emerging interventions. Longitudinal investigations are also required to standardize non-invasive diagnostic biomarkers, such as salivary metabolomics and tear proteomics, into routine, in-office testing.

## 5. Conclusions

Over the past decade, the ophthalmic care of Sjögren’s disease has undergone a fundamental transformation, evolving from palliative lubrication toward a structured, mechanism-directed treatment paradigm. Diagnostic evaluation is increasingly anchored in non-invasive modalities, including salivary metabolomics, regulatory non-coding RNA signatures, point-of-care tear MMP-9 testing, and salivary gland ultrasonography, which together permit earlier recognition of glandular dysfunction before irreversible fibrosis is established. Therapeutically, a stepwise clinical hierarchy now integrates preservative-free lubrication and tear conservation, targeted topical immunomodulation, trigeminal neurostimulation, mechanical shielding with cryopreserved amniotic membranes and customized scleral lenses, biological tear substitutes, and combined intense pulsed light with meibomian gland expression. The emergence of systemic B-cell–directed biotherapy, exemplified by ianalumab, signals a decisive shift from symptom control toward true disease modification of the underlying autoimmune exocrinopathy.

Nevertheless, the strength of this evolving framework is tempered by the predominance of evidence extrapolated from generalized dry eye cohorts rather than dedicated Sjögren’s disease populations. Validating these emerging diagnostics and therapeutics will require disease-specific Category 1 clinical trials, standardized non-invasive biomarkers, and longitudinal outcome data, ideally delivered through coordinated ophthalmic and rheumatologic care. Until such evidence matures, clinicians should individualize the escalating treatment hierarchy to disease severity, prioritizing early inflammation control and corneal protection to prevent vision-threatening complications.

## Figures and Tables

**Table 1:** Diagnostic, ocular surface and systemic interventions relevant to Sjögren disease (SjD)-associated dry eye, stratified by strength of disease-specific evidence.

Intervention/Modality	Primary Target/Mechanism	Study Cohort Profile	Evidence Category	Key Ocular Findings	Clinical Translation for SjD	Reference
Salivary gland ultrasonography (SGUS)	Parenchymal heterogeneity; hypoechoic foci; hyperechoic fibrous bands	SjD/suspected autoimmune cohorts	Category 1 (direct SjD evidence)	OMERACT score 2–3 correlates with severe glandular damage; parotid swelling with leukopenia and low C4 indicates high MALT lymphoma risk.	Non-invasive staging of structural exocrine damage; guides ultrasound-guided core-needle biopsy (CNB).	[13,19]
Point-of-care biomarkers and proteomics	Salivary metabolic analysis; H19 ICR hypomethylation; tear MMP-9	Human salivary and tear film models	Category 2 (mixed/model evidence)	Elevated salivary lactate, alanine and malate; point-of-care tear MMP-9 confirms active ocular surface inflammation.	Rapid, non-invasive confirmation of localized inflammatory and hypoxic exocrine cascades.	[12,18]
Large-diameter scleral lenses (SLs)	Pre-corneal liquid reservoir enclosure; complete evaporation blockage	Generalized DED and ocular surface disease	Category 3 (general DED, extrapolated)	≥8 h/day wear significantly reduces NEI corneal staining scores and improves BCVA; edge-aligned haptic toricity and polyethylene glycol (PEG) surface coatings mitigates midday fogging (MDF).	Continuous fluid shielding protects the desiccation-prone cornea and promotes active epithelial healing.	[9,15]
Amniotic membrane grafts	MMP-9 suppression; endogenous growth factor release	Refractory SjD and severe keratopathy	Category 1 (direct SjD evidence)	Rapid corneal re-epithelialization and anti-inflammatory relief; symptom relapse observed ~25 days post-dissolution.	In-office acute rescue bridge for severe corneal thinning, persistent defects or impending perforation.	[36]
Acoltremon 0.003% (Tryptyr)	Selective TRPM8 agonist on corneal cold thermosensory neurons	Generalized DED (phase 3 COMET-2/3 trials)	Category 3 (general DED, extrapolated)	Primary endpoint met with ≥10 mm Schirmer test increase at day 14; reduced SANDE/VAS burning and dryness scores.	Bypasses damaged secretomotor pathways to stimulate natural reflex tear secretion.	[10,21]
Water-free cyclosporine 0.1% (Vevye/Vevizye)	Calcineurin inhibitor in perfluorobutylpentane vehicle	Generalized DED	Category 3 (general DED, extrapolated)	Statistically superior in reducing total corneal fluorescein staining and conjunctival staining by day 29.	Enhanced ocular surface bioavailability for managing chronic autoimmune surface inflammation.	[25]
Lifitegrast 0.5%	LFA-1/ICAM-1 integrin antagonist	Generalized DED	Category 3 (general DED, extrapolated)	Selectively blocks T-cell binding to inflamed ocular epithelium, reducing local T-cell recruitment.	Suppresses upstream lymphocytic recruitment and ocular surface inflammatory drive in SjD.	[29]
Reproxalap	Reactive aldehyde species (RASP) modulator	Generalized DED (dry eye chamber model)	Category 3 (general DED, extrapolated)	Chemically neutralizes unbound aldehydes; increases tear output and reduces symptoms within 90 min of exposure.	Rapid anti-inflammatory rescue agent for managing acute ocular surface flares in SjD.	[26]
Hyaluronic Acid (HA) Therapeutics	Endogenous non-sulfated GAG biopolymers; provides non-Newtonian shear-thinning lubrication	Generalized DED, including subsets with aqueous deficient DED (ADDE), keratoconjunctivitis, and Sjögren’s Disease.	Category 1 (direct SjD evidence)	Significant improvements in TBUT, corneal/conjunctival OSS, and subjective symptom scores (OSDI, VAS); hypotonic formulations (0.18–0.4%) showed superior barrier and osmoprotective benefits over isotonic solutions in multiple cohorts.	Validated first-line baseline tear supplement for SjD; mitigates profound aqueous tear deficiency and reduces continuous blink-related shear stress on damaged epithelia.	[30]
Clyclosporine 0.1% in chondroitin sulfate emulsion (Klarity-C)	Calcineurin inhibitor (T-cell immunosuppression) delivered in a sulfated GAG vehicle (chondroitin sulfate) functioning as a membrane stabilizer and lubricating agent to reduce shear stress and enhance ocular bioavailability.	Generalized DED	Category 3 (general DED, extrapolated)	Statistically significant reduction in mean OSDI scores and mean corneal staining grade at 3 months; 50% of eyes achieved complete resolution of corneal staining (Grade 0) with no reported adverse events.	Well-tolerated, compounded alternative combining targeted T-cell calcineurin inhibition with GAG-mediated epithelial protection for chronic SjD-related keratopathy.	[32]
Heparin-like snail GAG (AFG)	Purified non-anticoagulant sulfated polysaccharide from *Achatina fulica*; promotes corneal epithelial proliferation/migration via K14, KI67, and PAX6; upregulates membrane mucins (*MUC1*, *MUC4*); downregulates pro-inflammatory cascades (IL-1β, TNF-α, IL-6, MMP-9, CD45, and macrophage infiltration).	In vitro human corneal epithelial (HCE) cels and in vivo benzalkonium chloride (BAC)-infused murine dry eye model	Category 3 (general DED, extrapolated)	Restored stratified corneal epithelial thickness, improved corneal fluorescein scores, increased tear secretion volume, and significantly suppressed ocular surface inflammatory cell infiltration.	Mechanistic proof-of-concept for treating severe autoimmune-driven epithelial breakdown, mucin deficiency, and chronic ocular surface inflammation without anticoagulant bleeding risks.	[31]
GlicoPro (Lacricomplex)	Standardized *Helix aspersa* snail mucus extract containing sulfated/unsulfated GAGs, scaffold proteins, and opiorphin; downregulates hyperosmolar pro-inflammatory markers (IL-1β, IL-6, IL-8, IL-20, TNF-α, MMP-9, MCP-1); reduces leukocyte chemotaxis and nociceptive signaling.	Generalized DED	Category 3 (general DED, extrapolated)	Significant reduction in hyperosmolar cytokines, MMP-9, and MCP-1 in vitro; statistically superior overall treatment effect vs. HPMC alone in reducing OSDI and SANDE scores, extending FBUT, and reducing corneal sub-basal dendritic cell density on IVCM.	Directly addresses the inflammatory vicious cycle and neurosensory discomfort of SjD by targeting matrix metalloproteinases (MMP-9) and suppressing corneal dendritic cell-mediated antigen presentation.	[33]
Perfluorohexyloctane (Miebo)	Non-aqueous, anti-evaporative tear film monolayer surrogate	DED patients undergoing cataract surgery	Category 3 (general DED, extrapolated)	Forms a protective surface monolayer; significantly reduces tear evaporation rate without blurred vision.	Conceptually addresses the co-existing evaporative component in mixed-etiology SjD tear volatility.	[34]
Lacripep (LOS-001)	Human lacritin C-terminal fragment; syndecan-1 receptor agonist	Human corneal epithelial models	Category 3 (general DED, extrapolated)	Mobilizes intracellular calcium; restores cell motility and accelerates corneal epithelial wound healing.	In vitro biological foundation for repairing chronic, non-healing corneal epithelial defects in SjD.	[27,35]
Autologous serum eye drops (ASED)/PRP	Endogenous growth factor delivery (EGF, TGF-β, NGF)	Severe/refractory SjD keratopathy	Category 1 (direct SjD evidence)	Both significantly improve TBUT and staining; PRP exhibits higher growth factor levels, aiding sub-basal nerve regeneration.	Biological epithelial repair and corneal re-innervation in severe, refractory SjD dry eye.	[37]
Ianalumab (VAY736)	Monoclonal BAFF-R antibody; dual ADCC B-cell depletion	SjD cohort (phase 3 THALASSA, NCT07621809; phase 3 NEPTUNUS-1/2)	Category 1 (direct SjD evidence)	Systemic depletion of pathogenic B cells; preserves exocrine gland function and reduces ESSPRI symptom burden.	Targeted systemic disease-modifying biotherapy addressing underlying SjD autoimmune dacryoadenitis.	[5,11,18,38]
Intranasal neurostimulation (varenicline, Tyrvaya)	Trigeminal parasympathetic pathway stimulation	Generalized DED and SjD subgroups	Category 2 (mixed/subgroup evidence)	Triggers endogenous nasolacrimal reflex tearing; improves Schirmer score, corneal staining and eye dryness without ocular contact.	Non-ocular secretomotor reflex activation for patients with severe ocular surface contact intolerance.	[22,23,24]
Intense pulsed light (IPL)	Broad-spectrum photothermal and anti-inflammatory eyelid therapy	Refractory SjD dry eye cohort	Category 1 (direct SjD evidence)	Significantly reduces OSDI score, extends TBUT, improves meibum quality and corneal staining; contraindicated in SLE.	Direct polytherapy for refractory SjD with co-existing meibomian gland dysfunction.	[39,41]
Vectored thermal pulsation (TearCare/LipiFlow)	Thermal evacuation of obstructed terminal meibomian ducts	Generalized meibomian gland dysfunction	Category 3 (general MGD, extrapolated)	Melts and expresses blocked meibum; significantly improves lipid layer thickness and dryness scores.	Potential adjunct strategy for managing the evaporative lipid-deficiency component in mixed SjD dry eye.	[40]

**Table 2:** Regulatory Status of Novel Interventions and Managements.

Intervention/Modality	Regulatory Status (FDA/EMA)	Availability
Salivary Gland Ultrasound (SGUS)	Standard diagnostic imaging modality (CE-marked/510 (k) cleared diagnostic ultrasound systems)	Established clinical diagnostic tool
Point-of-care biomarkers and proteomics	Most novel salivary/tear proteomic panels are research use only (RUS).	Standardized laboratory analysis limited to primary academic research institutions; select commercial assays (e.g., Bausch + Lomb’s Sjö panel) available in the United States.
Large-diameter scleral lenses (SLs)	FDA-cleared (e.g., PROSE 1994, gas-permeable materials); CE-marked medical devices in the European Union	Commercially available medical device. Requires fitting by contact lens specialists/corneal subspecialists in ophthalmic clinics.
Amniotic membrane grafts	FDA-cleared/approved biologic device (e.g., Prokera); regulated as human tissue products/CE-marked across Europe	Commercially available for in-office clinical application and grafting; accessible in specialized ophthalmology clinics.
Acoltremon 0.003% (Tryptyr)	FDA-approved (May 2025 for dry eye disease); not approved by the EMA	Commercially available by prescription in the United States; unavailable or restricted to clinical trials outside authorized markets.
Water-free cyclosporine 0.1% (Vevye/Vevizye)	FDA-approved (May 2023, as Vevye); European Commission-approved (October 2024, as Vevizye) for moderate-to-severe dry eye disease unresponsive to tear substitutes	Commercially available by prescription in the United States and, as Vevizye, in the European Union (marketed by Laboratoires Théa); regulatory applications pending in other markets.
Lifitegrast (Xiidra)	FDA-approved (July 2016); EMA application withdrawn in June 2020 (not approved in the European Union).	Commercially available by prescription in the United States; unavailable or restricted to clinical trials outside authorized markets.
Reploxalap	Not FDA- or EMA-approved	Investigational topical RASP inhibitor; under regulatory evaluation. Access strictly restricted to study protocols.
Hyaluronic acid (HA) therapeutics	FDA- and EMA-approved	Commercially approved by prescription across the United States, Europe, and globally. Available in eye care clinics.
Heparin-like snail GAG (AFG)	Not FDA- or EMA-approved	Currently restricted entirely to academic laboratory research. Not commercially available or accessible for human clinical use.
Cyclosporine 0.1% in chondroitin sulfate emulsion (Klarity-C)	Not FDA- or EMA-approved; unapproved compounded prescription in the United States.	Compounded prescription product available strictly via specialized ophthalmic compounding pharmacies (503A/503B facilities). Not available as standard retail pharmaceutical.
GlicoPro (Lacricomplex)	Not FDA-approved; EMA-regulated as CE-marked Class IIb medical device	Regionally approved commercial device (Europe). Commercially available as an ophthalmic solution in select European markets. Unavailable via commercial channels in the United States.
Perfluorohexyloactane (Miebo)	FDA-approved (May 2023 for dry eye disease); CE-marked as a medical device in the EU (e.g., EvoTears)	Commercially available as a prescription drug in the US and over the counter/medical device in several European jurisdictions. Broadly accessible.
Lacripep (LOS-001)	Not FDA- or EMA-approved	Investigational topical lacritin-derived peptide evaluated in Phase 2 clinical trials. Not commercially available.
Autologous serum eye drops (ASEDs)/PRP	Compounded biologic; not commercially FDA-approved (regulated under compounding guidelines); regulated under EU human tissue and blood directives.	Available as an unapproved, patient-specific compounded therapeutic. Primarily accessed through ophthalmic care centers and specialized compounding eye banks/pharmacies.
Ianalumab (VAY736)	Not FDA- or EMA-approved	Investigational anti-BAFF receptor monoclonal antibody; undergoing Phase 3 clinical evaluation (e.g., THALASSA trial) for Sjögren’s disease. Clinical trial access only.
Intranasal neurostimulation (varenicline, Tyrvaya)	FDA-approved (October 2021 for dry eye disease); not approved by the EMA	Commercially available by prescription in the United States; limited availability or pending approval internationally.
Intense pulsed light (IPL)	FDA-approved (April 2021 for dry eye disease due to MGD); CE-marked medical devices in the European Union	Commercially available clinical procedure; typically offered in specialized dry eye centers and private ophthalmic/optometric clinics (often out-of-pocket).
Vectored thermal pulsation (TearCare/LipiFlow)	FDA-cleared/510(k)-cleared medical device for MGD; CE-marked by Europe	Commercially available in-office procedures; largely limited to specialized dry eye practices and ophthalmology settings.

## Data Availability

No new data were created or analyzed in this study. Data sharing is not applicable to this article.

## Abbreviations

ADCC	Antibody-Dependent Cellular Cytotoxicity
ADDE	Aqueous-Deficient Dry Eye
ASED	Autologous Serum Eye Drops
BAFF-R	B-Cell Activating Factor Receptor
BCVA	Best-Corrected Visual Acuity
CAM	Cryopreserved Amniotic Membrane
CNB	Core-Needle Biopsy
DED	Dry Eye Disease
EV	Extracellular Vesicle
ESSDAI	Sjögren’s Syndrome Disease Activity IndexFRT: Fluid Reservoir Thickness
ICAM-1	Intercellular Adhesion Molecule-1
ICR	Imprinting Control Region
IFN-γ	Interferon-Gamma
IPL	Intense Pulsed Light
LFA-1	Lymphocyte Function-Associated Antigen-1
MALT	Mucosa-Associated Lymphoid Tissue
MDF	Midday Fogging
MGD	Meibomian Gland Dysfunction
MGX	Meibomian Gland Expression
MMP-9	Matrix Metalloproteinase-9
NEI	National Eye Institute
NSAID	Non-Steroidal Anti-Inflammatory Drug
OMERACT	Outcome Measures in Rheumatology Clinical Trials
OSDI	Ocular Surface Disease Index
PEG	Polyethylene Glycol
PRP	Platelet-Rich Plasma
RASP	Reactive Aldehyde Species
RCT	Randomized Controlled Trial
SANDE	Symptom Assessment in Dry Eye
SGUS	Salivary Gland Ultrasonography
SjD	Sjögren’s Disease
SL	Scleral Lens
TBUT	Tear Breakup Time
TNF-α	Tumor Necrosis Factor-Alpha
TRPM8	Transient Receptor Potential Melastatin 8
VAS	Visual Analog Scale
